# 

**Published:** 1907-02-15

**Authors:** 


### ANNOUNCEMENTS.

Consolidation of The Fraternal Dental Society and The Society of Dental Science of St. Louis.— At joint meeting of The Fraternal Dental Society and The Society of Dental Science of St. Louis, held December

18, 1906, a consolidation of the two societies was effected, the society to be known in the future as The St. Louis Society of Dental Science.

The officers and committees for the ensuing year are: D. O. M. LeCron, President; Richard Summa, Vice-President; Clarence O. Simpson, Secretary; W. E. Brown, Treasurer. Executive Committee—W. L. Whipple, E. E. Haverstick, Herman F. Cassel. Advisory Council—George A. Bowman, A. H. Fuller. Adam Flickinger, Wm. Conrad, Burton Lee Thorpe, Edward H. Angle, E. P. Dameron.

KENTUCKY STATE DENTAL ASSOCIATION.—The next annual meeting of the Kentucky State Dental Association will convene at Louisville, Ky., May 20, 21, and 22, 1907. We anticipate a most interesting and profitable meeting. A cordial invitation is extended to the profession.

W. M. RANDALL,
*Secretary.*

THE GARHART DENTAL MANUFACTURING COMPANY SELLS OUT.—The Harvard Company, of Canton, Ohio, has purchased the plant of the Garhart Dental Manufacturing Company, of Indianapolis, Ind., and will incorporate that company into the Harvard.

JAMESTOWN DENTAL CONVENTION.—The Jamestown Dental Convention, to be held under the auspices of the Jamestown Exposition Company, the Southern Branch of the N. D. A., and the Virginia State Dental Association, will convene at Norfolk, Va., September 10 to 12, 1907. The Jamestown Exposition Company has appointed the below named gentlemen as a Committee on Organization, to elect officers in advance of the meeting, to appoint all committees, to finance the meeting, and to bring it to a successful termination.

The Committee on Organization has appointed Dr. Clarence J. Grieves, Baltimore, Md., general chairman of the Clinic Committee and Supervisor of Clinics.

A number of well-known men will assist him on the General Committee. State clinic chairmen have been selected from every State in the Union. The clinics are to be the principal feature of the convention, and it is expected to bring about the largest and most instructive dental clinics ever held. A surgical clinic will also be held under the supervision of Dr. L. M. Cowardin, Richmond, Va. The other members of this committee are J. Y. Crawford, Nashville. Tenn., and A. G. Friedrichs, New Orleans, La. Dr. F. W. Stiff, Richmond, Va., is general chairman of the Membership Committee.

Assistant state chairmen have been appointed from every State in the Union. Already membership fees are being sent in, and the promise is for the largest gathering of dentists ever held. Only five essays will be read at the convention, one by Prof. W. D. Miller, another by Prof. G. V. Black, and the other three by well-known southern dentists.

Several exhibits of much interest to the profession will be held under the auspices of the convention; among them the dental manufacture exhibit in charge of Dr. John W. Manning, chairman, Norfolk, Va,; a comparative anatomy exhibit, in charge of Dr. W. M. Bebb, chairman, Los Angeles, Cal., which exhibit will consist of three thousand comparative anatomy specimens, and also numerous other collections of interest; a dental historical exhibit, consisting of ancient instruments, operative and prosthetic work, books and photographs, under the chairmanship of Dr. Wm. H. Trueman, Philadelphia, Pa.; the orthodontia exhibit, showing a large collection of models, etc., under the chairmanship of Dr.

H. E. Kelsey, Baltimore, Md. The U. S. Naval dental exhibit, showing 3000 charts of the mouths of midshipmen, under the chairmanship of Dr. John S. Marshall, San Francisco, Cal., will also show the equipment, method of keeping records, etc., used by the dental corps.

A full list of the various officers, who are to be elected in advance by the Committee on Organization at their next meeting in February, 1907, and of the committees, will appear in due time in the various dental journals. The Committee of Organization is expected to select officers in advance in order that the officers may be prepared for their duties before the actual meeting of the convention.

A cordial invitation is extended to all reputable members of the profession to become members of this convention, and to assist the Committee on Organization in bringing about one of the best, if not the best, dental meeting ever held.

The Exposition itself offers an excellent opportunity for the busy practitioner to take a delightful vacation, see the wonderful historical, naval and military exhibits at the Exposition, and also to participate in this meeting. The membership fee, which is $5.00, should be sent to Dr. F. W. Stiff, treasurer, 600 East Grace St., Richmond, Va.

*Committee of Organization:* Burton Lee Thorpe, St. Louis, Mo., Chairman; H. Wook Campbell, Suffolk, Va., Secretary; F. W. Stiff, Richmond, Va., Treasurer; R. H. Walker, Norfolk, Va.; Thos. P. Hinman, Atlanta, Ga.; J. E. Chace, Ocala, Fla.; Clarence J. Grieves, Baltimore, Md.

For further information address

H. W. CAMPBELL, *Sec'y*,
Suffolk, Va.

---

MARRIED. LODGE-TOWNSEND.—Dr. E. B. Lodge, the well-known dentist of Cleveland, was married December 27th, 1906, to Miss Martha Townsend, at Odessa, Delaware.

MARRIED. DENNIS-JENKINS.—Dr. C. P. Dennis, of Portsmouth, Ohio, and Miss Anna Jenkins, of Williamsburg, Ohio, were married November 28th, at Batavia, Ohio.

—✦—

SOUTHERN CALIFORNIA DENTAL ASSOCIATION.—The tenth annual session of the Southern California Dental Association will be held in Los Angeles, May 6, 7 and 8, 1907, at the same time that the Imperial Council of Mystic Shrine meets here, and all members of the dental profession contemplating visiting southern California at that time will confer a favor upon the association by notifying the secretary.

CHAS. M. BENBROOK,
455 S. Broadway, Los Angeles, Cal.

—✦—

AMERICAN DENTAL SOCIETY OF EUROPE.—The American Dental Society of Europe will hold its next annual meeting in Rome, Italy, on March 29 and 30, and April 1. A very cordial invitation is extended to members of the profession to be present.

As it is the first meeting of the society in the Eternal City, it is hoped it may be the most enjoyable one in its history.

J. W. GALE, *Hon. Sec'y*,
79 Hohenzollern-Ring, Cologne (Rhine), Ger.

